# Integrating Plastic Waste into Concrete: Sustainable Solutions for the Environment

**DOI:** 10.3390/ma17143408

**Published:** 2024-07-10

**Authors:** Maria Concetta Oddo, Liborio Cavaleri, Lidia La Mendola, Hassan Bilal

**Affiliations:** Department of Engineering, University of Palermo, 90128 Palermo, Italy

**Keywords:** plastic waste, concrete, plastic pollution, waste recycling, mechanical performance

## Abstract

Plastic waste management has received significant attention in recent decades due to the urgent global environmental crisis caused by plastic pollution. The versatile and durable nature of plastic has led to its widespread usage across various sectors. However, its nonbiodegradable nature contributes to unsustainable production practices, leading to extensive landfill usage and posing threats to marine ecosystems and the food chain. To address these environmental concerns, numerous challenges have been recently addressed through investigating alternative approaches for disposing of plastic waste, with the construction sector emerging as a promising option. Incorporating plastic waste materials into concrete not only offers economic benefits but also provides a valid alternative to conventional disposal methods. This paper presents the results of different experimental studies, some of them available in the literature and others new, discussing the feasibility of integrating plastic waste into concrete and its impact on mechanical properties. The influence of different sizes, natures, treatments, and percentages of plastic waste in the concrete mixtures is dealt with in order to provide further data for helping to understand the nonunivocal results in the literature, under the conviction that only further observations can help to understand the mechanics of concrete with plastic aggregates. The experimental investigation highlighted that one parameter that is better than others and can be considered to compare different experimental investigations is the variation in weight (due to the effective volume of plastics in the mix), determining a sort of increasing of porosity that degrades the mechanical characteristics. However, this seems inconsistent in some cases. Therefore, the need for further research is highlighted to refine production methods and optimize mix designs.

## 1. Introduction

In recent decades, plastic waste has emerged as a growing and pressing concern. Originating from the introduction of synthetic polymers in the 1940s [1], plastics have revolutionized industries and daily life, offering unique properties such as light weight, strength, corrosion resistance, durability, versatility, and cost-effectiveness [2]. Consequently, plastic has found applications in almost every sector, including packaging, the food and beverage industry, electronics and electrical appliances, textiles and clothing, healthcare and medical devices, agriculture, construction and building materials, and the automotive industry. Plastic objects are typically designed for single-use purposes, resulting in their disposal within a short timeframe [3]. Consequently, this practice has led to the rapid accumulation of large quantities of plastic waste. Plastic waste disposal began to be observed in 1970, but the importance of the presence and impacts of plastic waste was only recognized in the early 21st century after plastic waste disposal had persisted uncontrolled for a significant period. Recent estimations suggest that plastics constitute a substantial fraction, approximately 10–13%, of municipal solid waste globally, with an annual production exceeding 1 billion tons [4]. 

The United Nations Environment Assembly reports that of the seven billion tons of plastic waste generated globally so far, less than 10% has been recycled. Millions of tons of plastic waste are lost to the environment. It is estimated that 75 to 199 million tons of plastic is currently found in our oceans. The amount of plastic waste entering aquatic ecosystems could nearly triple from 9–14 million tones per year in 2016 to a projected 23–37 million tons per year by 2040 [5].

Plastic materials are predominantly nonbiodegradable and can persist in the environment for extended periods—ranging from 20 years for items like plastic bags to up to 600 years for materials such as fishing lines [1]. This persistence poses a grave threat to ecosystems and human well-being [6].

A direct consequence of the plastic waste persisting in the environment is the generation of microplastics, which are small particles or fibers released into the environment through processes like photodegradation and mechanical breakdown. These tiny plastic particles can be ingested by marine life and terrestrial animals, polluting the food chain [7]. Moreover, improper disposal methods such as landfilling, incineration, and ocean dumping [8,9] can lead to the release of greenhouse gases and toxic pollutants into the environment, thereby causing pollution of the soil, marine environments, and air.

The increasing awareness of the environmental and societal impacts of plastic waste has garnered the interest of the scientific community, prompting efforts to address these pressing issues [10]. Innovative technological solutions are being developed, encompassing chemical recycling and pyrolysis methods aimed at converting plastic waste into valuable feedstocks for the creation of new products [11,12], plastic-degrading enzymes that target specific types of plastics [13], and waste-to-energy technologies designed to convert plastic waste into heat, electricity, or fuels through processes like gasification or anaerobic digestion [14]. 

Among these advanced technologies, directly recycling and reusing of plastic waste by integrating it into building materials are gaining significant attention [15,16]. Indeed, recycling plastic waste in construction materials can yield multiple benefits, including enhancing the sustainability of the construction industry and maximizing the advantages for both the circular economy and the environment. Notably, incorporating plastic waste into concrete represents a promising solution for reducing the consumption of natural aggregates and effectively managing plastic waste that would otherwise end up in landfills or oceans [17], contributing to environmental degradation.

Research findings from the literature reveal that the global demand for natural construction aggregates peaked at 45 billion tons in 2017, with projections indicating a further escalation in the upcoming years [18]. The potential substitution of approximately 10% of normal aggregates with plastic aggregates could save over 820 million tons from inappropriate disposal in the environment [19]. However, the introduction of plastic waste in concrete needs careful consideration to minimize adverse impacts while optimizing benefits. 

Generally, the integration of plastic waste into concrete can result in a decrease in certain mechanical properties, such as compressive and flexural strength, steel–concrete bond, and workability. When substituting up to 10% of the sand in concrete with plastic waste, the reduction in compressive strength may be minimal [20,21]. However, this effect becomes more pronounced with higher proportions of plastic waste aggregates (>15%) [22]. Various factors, including the nature, type, size, and treatment of plastic aggregates, influence the final performance of concrete [19]. Additionally, employing appropriate methods for concrete mixture preparation is essential for reducing the voids and optimizing the final result. 

Despite the decrease in compressive strength, concrete containing plastic waste aggregates offers significant advantages. It presents a practical solution for constructing nonstructural or lightweight elements like pavement blocks and slabs, where low density and moderate strength are essential [23]. Moreover, the inclusion of plastic waste as fiber reinforcement can improve the failure behavior by limiting crack propagation and enhancing ductility [23,24,25]. These concrete mixtures have found applications where flexibility and deformation capacity are fundamental properties, such as in the composite materials utilized for structural retrofitting applications. Additionally, an important aspect lies in the improvements in thermal and sound insulation properties compared to those of traditional concrete mixtures [16]. Recent studies have demonstrated that concrete incorporating plastic waste as filler material exhibits a higher absorption capacity, with noise reduction values up to 57% greater [26,27]. These acoustic insulation characteristics make concrete with plastic aggregates suitable for applications in noise-sensitive environments like hospitals or areas near heavily trafficked roads. Moreover, it was noticed that the usage of filler materials and plastic waste as 100% natural aggregate led to the enhancement in the thermal conductivity by 49% compared with that of natural lightweight aggregate concrete [16]. This indicates a significant improvement in thermal insulation, offering potential energy efficiency benefits in building development.

This literature review underscores a recent trend where significant efforts have been focused on substituting natural aggregates in concrete mixes with plastic aggregates. However, this substitution poses several challenges due to the unavoidable differences in characteristics between plastic and natural aggregates. In spite of numerous studies that have explored this topic, the results in the literature from different experimental campaigns are not consistent. This means that the various mechanisms involved are not completely clear, and further experimental observations are needed. In the present study, the results of some experimental campaigns aimed at evaluating the mechanical characteristics of concrete incorporating plastic materials in different forms for different applications are discussed. In each case, the final goal is the recognition of potential solutions for sustainably managing plastic waste. 

This paper focuses on the principal characteristic required for concrete, which is compressive strength, trying to provide a set of experimental results for comparison with the results in the literature, which are inconsistent as mentioned above, and to capture the incompletely clear mechanisms that influence the variation in the strength of concrete when plastic aggregates are used or at least to understand the mechanisms that limit the degradation in the mechanical characteristics. Tests for assessing plastic aggregate debonding properties and the consequent durability have not been proposed, even if it is recognized how important this issue is for concrete performance. 

Various sizes, percentages, and types of plastic wastes used as aggregates, subjected or not to previous treatments, have been incorporated into different concrete control mixes, including lower- and higher-strength concrete, as well as pervious concrete mixes enhanced with silica fume or fly ash.

This paper focuses on the mixes, leaving any issue regarding the durability and the interaction with steel bars in the case of structural applications to future developments.

## 2. Experimental Campaigns 

The experimental campaigns involved compressive tests on concrete cubes, both with and without plastic aggregates, to evaluate the strength variations across different concrete formulations, plastic types, and percentages.

In detail, the impact of different percentages and types of plastic waste aggregates—i.e., light-blue plastic flakes, plastic flakes, and plastic granules—on two distinct ordinary concrete mixes characterized by differing strengths was reported: one having low strength (C1) and the other having high strength (C2). Those data were integrated with the results of an experimental campaign in which the influence of incorporating fibers obtained from plastic waste into concrete blends, so-called “pervious” concrete, was observed. The referenced pervious concrete mix comprised only cement, sand, and coarse aggregate (PC). The blends with fibers included superplasticizer additives to improve the workability. In the case of pervious concrete, the effect of a partial substitution of cement with silica fume (PC-SF) or fly ash (PC-FA) was also studied. 

Experimental results from other authors were also used for comparison and for underlining the inconsistencies in the literature.

### 2.1. Materials

Ordinary concrete mixes C1 and C2 were formulated using Portland limestone cement Type 32.5, along with quarry sand whose grains had a maximum size of 2 mm and crushed rock coarse aggregate with a maximum size of 20 mm. Additionally, three types of plastic waste aggregates—light-blue plastic flakes (P1), plastic flakes (P2), and plastic granules (P3)—were incorporated into these concrete mixes. These plastic aggregates varied in size, composition, and treatment. 

**Figure 1:**
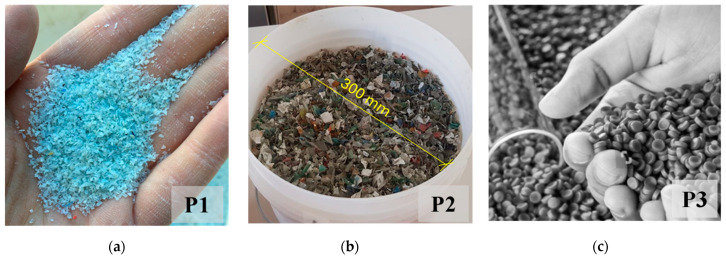
Plastic waste aggregates: (**a**) light-blue plastic flakes, P1; (**b**) plastic flakes, P2; (**c**) plastic granules, P3.

Light-blue plastic flakes (P1), shown in Figure 1a, were derived from shredded and washed recycled plastics, primarily composed of polyethylene (PE), polypropylene (PP), and polyvinyl chloride (PVC). They might have contained some impurities and residual contaminants from the recycling process, such as metal particles, wood fragments, and polyolefins. These flakes were characterized by a maximum size of 2 mm and a bulk density of 610 kg/m^3^. Plastic flakes (P2), shown in Figure 1b, were obtained by shredding various types of plastic bottles, primarily composed of polyethylene terephthalate (PET), PE, and PVC. These flakes underwent no specific treatment or washing process and were irregularly shaped, with a maximum size of 20 mm. They were characterized by a bulk density of 400 kg/m^3^. Finally, plastic granules (P3), shown in Figure 1c, were produced through a process of grinding, washing, fusion, and extrusion of PE and PP plastics. They were extruded into quasicylindrical shapes with a maximum size of 10 mm and had a bulk density of 580 kg/m^3^.

**Figure 2:**
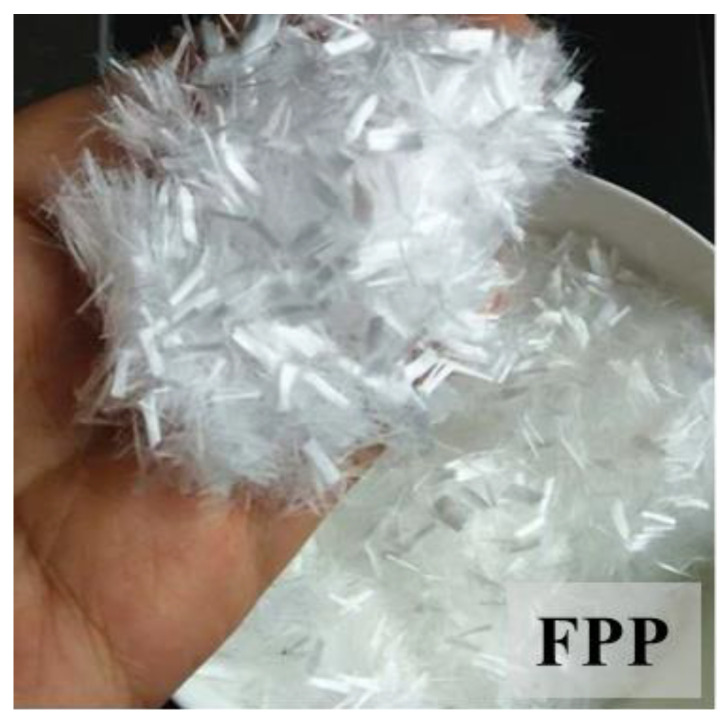
Polypropylene fibers, FPP.

Pervious concrete mixes, including PC, PC-SF, and PC-FA, were produced using Portland cement with a 42.5-grade strength as a binder, in addition to fine sand, coarse aggregates ranging in size from 4.75 mm to 9.5 mm, and polycarboxylate superplasticizers to enhance the workability of the mixtures. In PC-SF, a portion of the Portland cement was replaced with silica fume, characterized by spherical particles with a diameter of less than 1 μm and a specific surface area of 17.30 m^2^/g. Conversely, in PC-FA, a portion of the cement was substituted with class II fly ash, characterized by a high percentage, about 56.16%, of silica (SiO_2_). Fibers of polypropylene (FPP) with a length of 9 mm, as depicted in Figure 2, were introduced into all three pervious concrete mixes. The fibers were obtained by crushing, cutting, and washing various packages. Then, drying and melting preceded spinning. The true density of the PP fibers was approximately 900 kg/m^3^. 

### 2.2. Mix Proportions

All the concrete mixes included in the experimental program are summarized in Table 1, with each mix designated according to the format X_Y_N, where X represents the type of mix: C1 and C2 are used for ordinary concrete, and PC, PC-SF, and PC-FA for pervious concrete; Y indicates the type of plastic aggregates, categorized as P1, P2, P3, and FPP for light-blue plastic flakes, plastic flakes, plastic granules, and plastic fibers, respectively; N indicates the percentage by bulk volume of plastic aggregates added to the reference mix (0%, where used, refers to the concrete reference mixes). In the manufacturing of the mixes with plastics, the plastics were added to the concrete with the reference mix, that is, to each component of the concrete. The consequence was that the true volume of plastics in one cubic meter was lower than that nominally used for the mixes themselves. 

The control concrete mixes, C1 and C2, utilized in combination with light-blue plastic flake aggregates (P1), were characterized by the following percentages by weight: 14% and 18% cement, 29% and 27% sand, and 49% and 47% coarse aggregates, respectively. Both mixes had a water–cement ratio (*w*/*c*) of 0.5. In the case of the low-strength mix, C1, P1 aggregates were added according to the following percentages by volume, 10%, 20%, and 30%. For the high-strength mix, C2, P1 aggregates were incorporated at percentages by bulk volume of 2%, 4%, 6%, and 30%. In this case, low and high percentages of plastics were chosen to be added to determine if there was a different effect in the two cases in terms of a decrease in strength. The value of 30% plastics was compared directly with the reference mix with values of 2%, 4%, and 6% to capture possible variations in the slope of the curves expressing the mechanical characteristics vs. the percentage of plastic. Effectively, different variations in the characteristics of concrete were identified (higher for low quantities of plastic) as will be shown hereinafter.

The reference concrete mix, C1, utilized in combination with plastic flake aggregates (P2), was characterized by a *w*/*c* ratio of 0.6 (to guarantee sufficient workability after plastics’ addition) and the following percentages by weight: 16% cement, 39% sand, and 36% coarse aggregates. The P2 aggregates were added to the low-strength mix C1 at 10% and 20% percentages by bulk volume. 

The reference control mix, C2, combined with plastic granule aggregates (P3), was characterized by a *w*/*c* weight ratio of 0.5. The following percentages by weight, 18% cement, 25% sand, and 48% coarse aggregates, were used. The P3 aggregates were added to the higher-strength concrete mix C2 at percentages by bulk volume of 10%, 20%, and 30%. 

**Figure 3:**
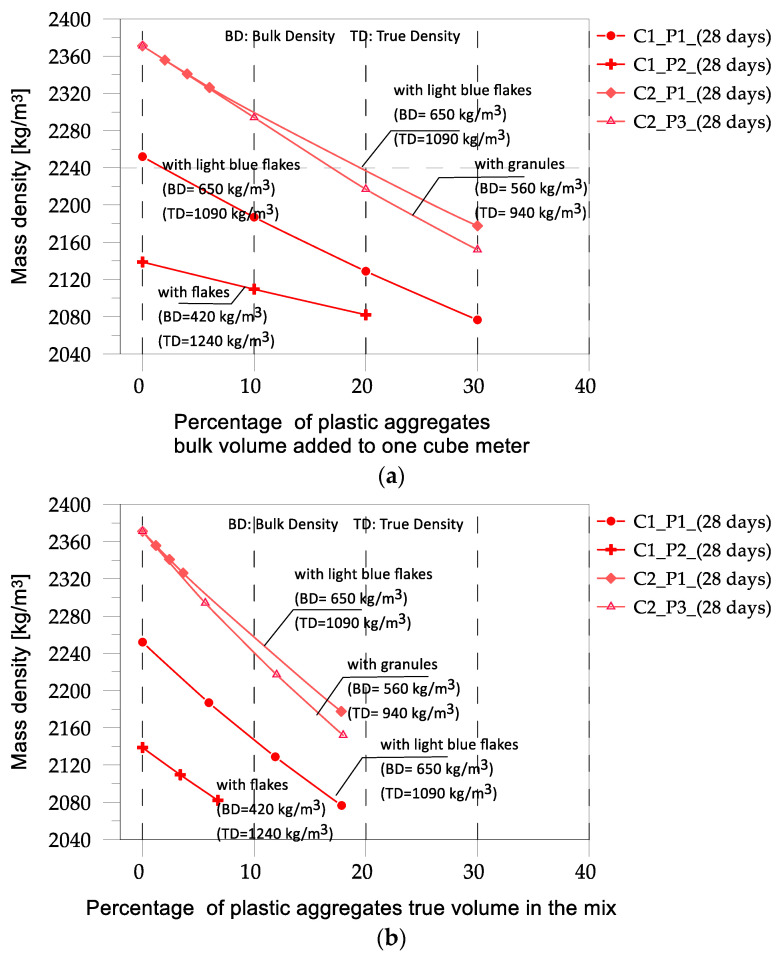
Mass densities correlated to the bulk volumes (**a**) and the true volumes (**b**) of plastic aggregates.

In Figure 3, the mass densities of the different mixes are reported, depending on both the added bulk volume of plastic aggregates and the amount of plastic in one cubic meter in terms of percentage of true volume. 

The control pervious concrete mix, PC, combined with polypropylene fibers (FPPs) at a volume fraction of 0.2% (the true volume was considered in the calculation of this rate), was characterized by the following weight proportions: 17% cement, 4% sand, and 74% coarse aggregates. A *w*/*c* weight ratio of 0.5 was maintained. To enhance the consistency and workability of the mix, a polycarboxylate superplasticizer was at 1.25% of the weight of the cement. 

The control pervious concrete mixes PC-SF and PC-FA, incorporating polypropylene fibers (FPPs) at a volume fraction of 0.2% (corresponding to 1.8 kg/m^3^), were characterized by the following weight proportions: 16% cement, 4% sand, and 74% coarse aggregates, with a w/c ratio of 0.5 and superplasticizer added at 1.25% of the weight of cement. Additionally, in the PC-SF and PC-FA mixes, silica fume and fly ash, respectively, replaced 10% of the weight of the cement. 

The function of the fibers in concrete is to limit the Poisson effect. This increases the compressive strength, delaying the formation of cracks along the direction of loading. The percentage of fibers was chosen to guarantee the sufficient workability of the pervious concrete, which needed to be supported by the use of a superplasticizer. 

Figure 3b gives evidence of an elementary fact: plastics with close to the true mass density caused similar variations in the mass density of the concrete containing them. This fact is not clear from Figure 3a, where the bulk volume percentages are reported, where different slopes of the mass density vs. plastic bulk volume percentage curves can be observed. On the other hand, the true plastic mass density is a much more reliable indicator of the correlation with the variation in the concrete’s mechanical characteristics, because it gives a real measure of the weak component of concrete. 

### 2.3. Testing Procedure

**Figure 4:**
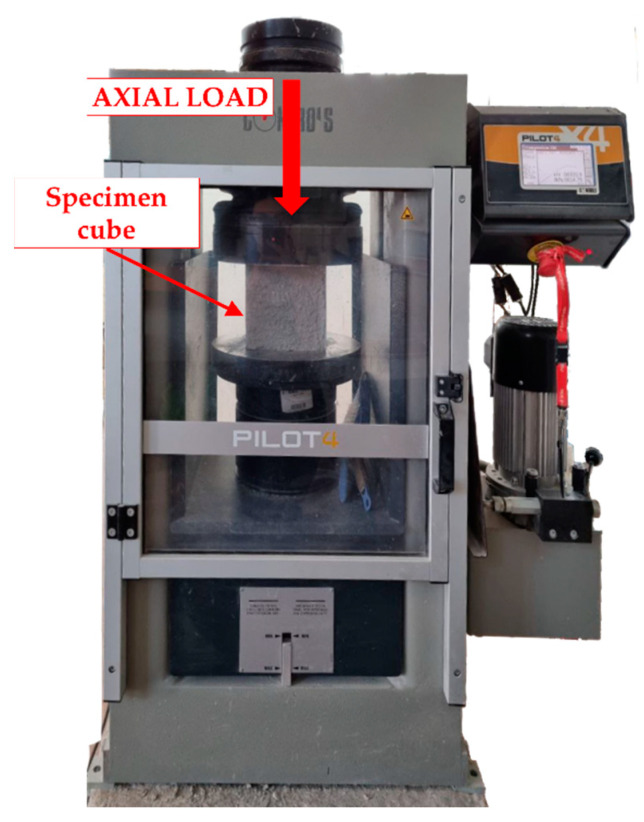
Test setup configuration.

Monotonic compressive tests were carried out on specimen cubes according to UNI EN 12390-4:2002 [28] and UNI EN 12390-3:2009 [29]. Concrete mixes C1 and C2, with and without plastic aggregates, were tested after curing periods of 7, 14, and 28 days under controlled environmental conditions of 20 ± 2 °C and 95% relative humidity. Similarly, pervious concrete mixes, denoted as PC, PC-SF, and PC-FA, were subjected to compressive tests after curing for 7, 28, and 90 days. Each concrete mix was cast into molds measuring 150 × 150 × 150 mm and compacted using a vibrator. Three specimens were prepared for each concrete mix with different curing times to ensure accurate and reliable test results. Figure 4 shows the compression machine used for the compression tests.

The mass density was noted each time. Furthermore, for the types of mixes investigated, the tensile strength derived by the split test and elastic modulus in compression was also experimentally obtained and is here reported for the sake of completeness, despite the minor relevance with respect to the compressive strength, which was the mainly focus in this study.

## 3. Experimental Results

The average experimental results in terms of compressive strength for concrete mixes C1 and C2 are listed in Table 2, alongside the normalized compressive strength values. The latter were calculated by dividing the compressive strength of the concrete mixes with plastic aggregates by the strength of the corresponding reference control mix.

**Figure 5:**
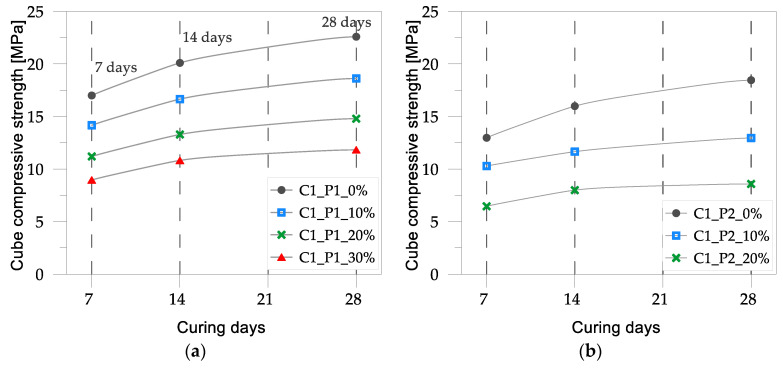
Cube compressive strength vs. curing for concrete mix C1 with varying percentages of plastic aggregates: (**a**) light-blue plastic flakes, P1; (**b**) plastic flakes, P2.

**Figure 6:**
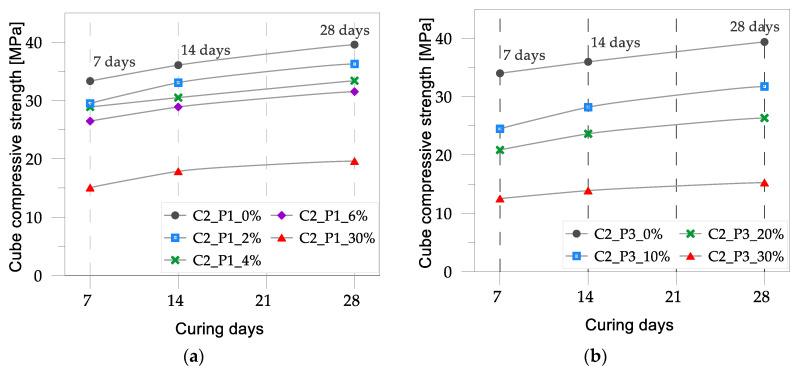
Cube compressive strength vs. curing days of concrete mix C2 with varying percentages of plastic aggregates: (**a**) light-blue plastic flakes, P1; (**b**) plastic granules, P3.

**Figure 7:**
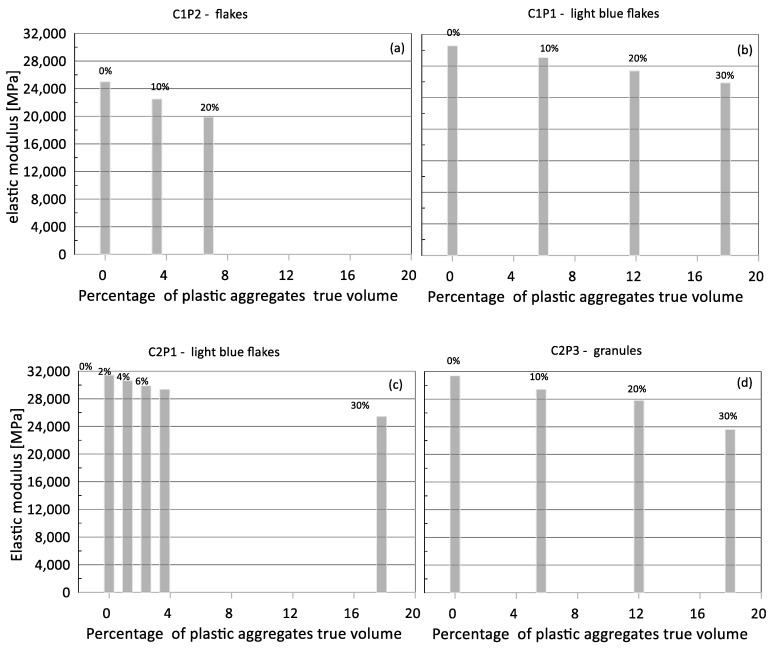
Elastic modulus of compression of the different mixes vs. true percentage of plastic aggregates by volume in the mix: (**a**) mix C1P2, (**b**) mix C1P1, (**c**) mix C2P1, (**d**) mix C2P3.

**Figure 8:**
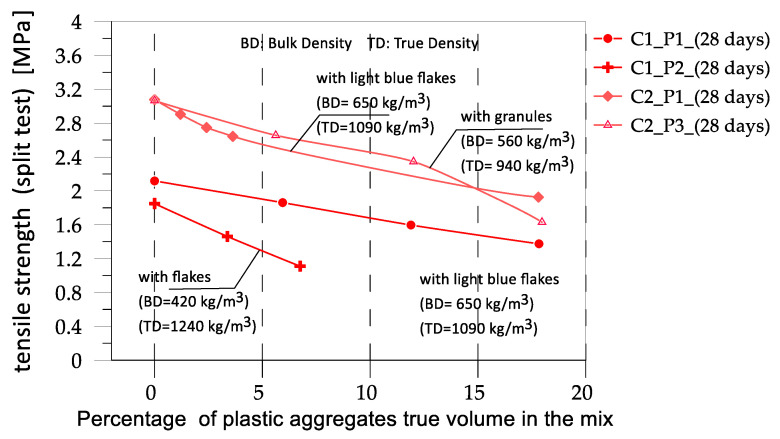
Tensile strength of the different mixes vs. the true percentage of plastic aggregates by volume.

In Figure 5 and Figure 6, the cube compressive strength values are plotted against the curing period for concrete mixes C1 and C2, respectively. In Figure 7 and Figure 8, the tensile strength and elastic modulus are presented. As expected, both concrete mixes exhibited a strength that gradually increased with the curing duration. An inverse relationship was observed between the percentage of plastic aggregates and the compressive strength, with higher percentages resulting in lower strength.

In general, the reduction in strength for concrete mixes with lower percentages (10%) of plastic aggregates was relatively moderate: approximately 17% for concrete mix C1 with light-blue plastic flakes (P1), 26% for concrete mix C1 with plastic flakes (P2), and 23% for concrete mix C2 with plastic granules (P3). However, as the percentage of plastic aggregates increased, the decrease in strength became more pronounced. With 30% plastic aggregates added to the mix, the decline in strength was substantially higher, ranging from about 47% (for concrete mix C1 with light-blue plastic flakes) to approximately 62% (for concrete mix C2 with plastic granules).

The most significant impact on concrete strength was observed when plastic flakes (P2) were added to control mix C1, with a 20% inclusion rate resulting in a substantial 51% reduction in strength. The lowest impact was observed when light-blue plastic flakes (P1) were added to the control mix. In this case, the loss in strength was relatively contained, especially for a lower percentage of plastic flakes, as in concrete mix C2, where a 9% decrease in strength was observed at a 2% inclusion rate.

The average experimental results in terms of compressive strengths at different curing time points (7, 28 and 90 days) for the pervious concrete mixes, PC, PC-SF and PC-FS, are listed in Table 3, alongside the normalized compressive strength values.

**Figure 9:**
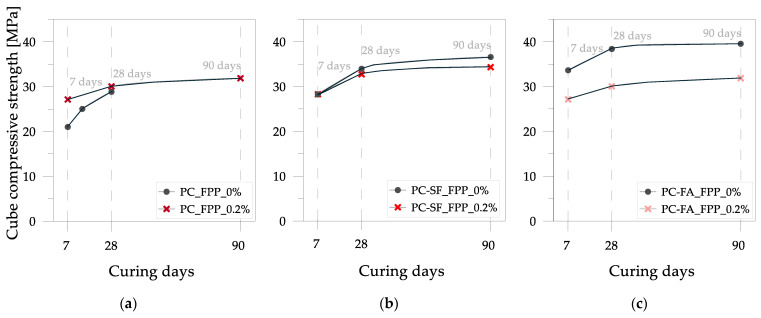
Cube compressive strength vs. curing time for pervious concrete mixes: (**a**) mix PC; (**b**) mix PC-SF with silica fume; (**c**) mix PC_FA with fly ash.

**Figure 10:**
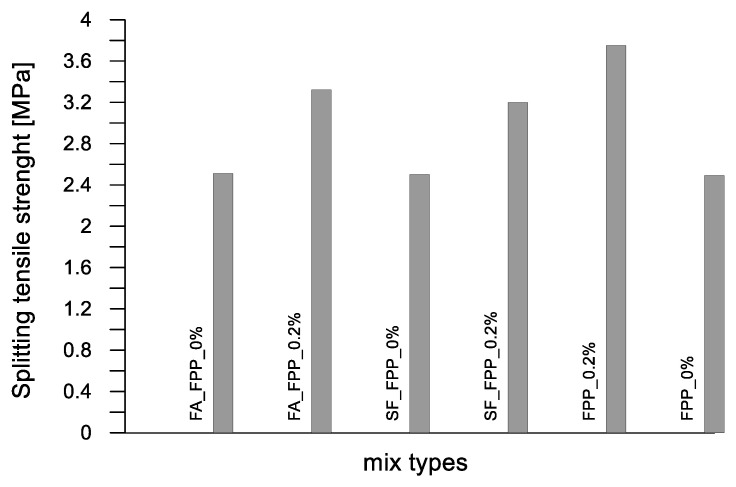
Splitting tensile strengths of pervious concrete mixes.

**Figure 11:**
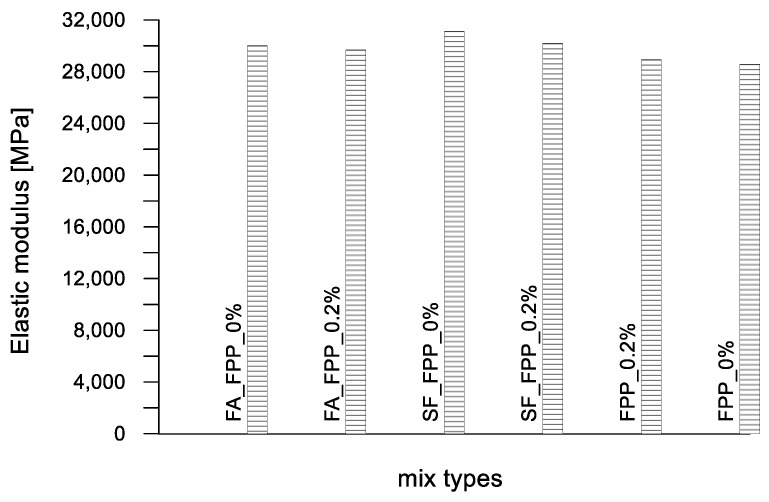
Elastic moduli of compression of pervious concrete mixes.

The cube compressive strength values are plotted against the curing days in Figure 9. Furthermore, the tensile strengths and elastic moduli are presented in Figure 10 and Figure 11. In this case, the mixes are characterized by a substantially constant mass density because of the negligible influence of the weight of fibers added to the unitary volume (2130 kg/m^3^). Contrary to what was obtained for the ordinary concrete mixed with plastic granules, sand and flakes, the inclusion of plastic fibers in pervious concrete mix PC led to an increase in strength for curing periods of less than 28 days. This increase was particularly noticeable at 7 days of curing, with the trend stabilizing at 28 days, where the compressive strength values closely aligned (28.8 MPa for the control mix PC_FPP_0% and 30.0 MPa for the mix with plastic fibers PC_FPP_0.2%). A slight loss of strength (approximately 3%) was observed in the pervious concrete mixes with silica fume and plastic fibers (PC-SF_FPP_0.2%) compared to the control mix (Ref. PC-SF_FPP_0%). This decrease in strength became more pronounced (about 10%) for the pervious concrete mixtures with fly ash and plastic fibers (PC-FA_FPP_0.2%).

Clearly, the reduction in mechanical performance of concrete due to the addition of plastics was more evident for mixes C1 and C2, where the plastic fibers’ inclusion rate was higher than that introduced into the pervious concrete mixes. However, introducing a high percentage of plastic fibers to the pervious concrete is not easy due to the segregation that arises during the mixing process.

## 4. Comparison and Discussion

**Figure 12:**
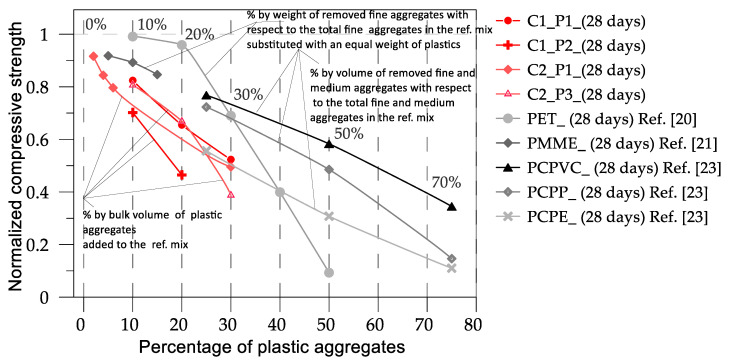
Comparison between the normalized compressive strengths obtained in the present study and data from the literature [20,21,23] referring to the reported plastic percentages.

**Figure 13:**
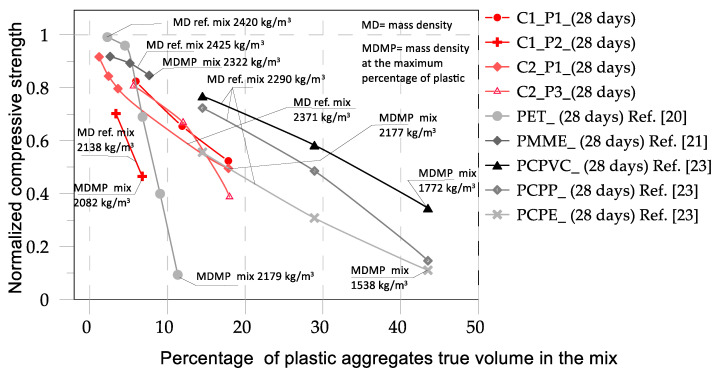
Comparison between the normalized compressive strengths obtained in the present study and data from the literature [20,21,23] referring to the true percentage of plastic by volume.

**Figure 14:**
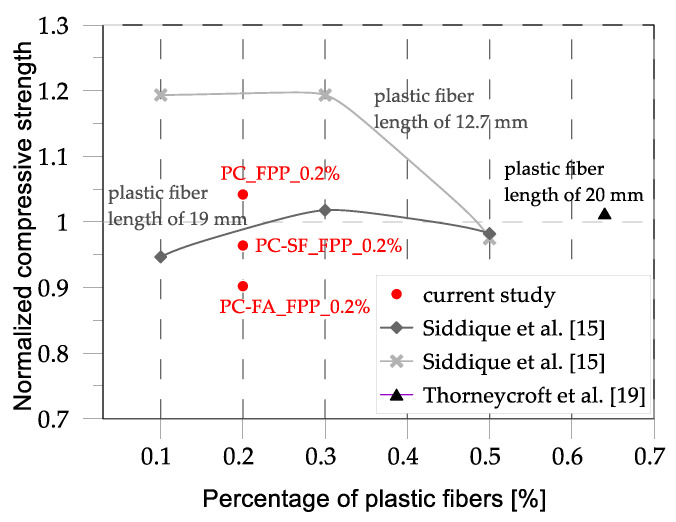
Comparison between the normalized compressive strengths obtained in the present study and data from the literature [15,19].

Comparisons among the results of the current experimental study and the available data in the literature are presented in Figure 12, Figure 13 and Figure 14. Specifically, Figure 12 compares the results of concrete mixes C1 and C2 with the data available in [20,21,23]. In Figure 13, the results of pervious concrete mixes PC, PC-SF, and PC-FA are compared with the data available in [15,19]. In Figure 12, the comparisons are plotted in terms of normalized compressive strength vs. the percentage of plastic aggregates as declared in [20,21,23]. Figure 13 displays the comparisons after having transformed the percentages of plastics declared in the previous studies in true percentages by volume. In Figure 13, the mass densities of the most significative reference mixes are also depicted with the mass densities of the mixes from each group of studies that characterized the maximum percentage of plastic aggregate. In Figure 12, the physical characteristics of the modified mixes in terms of normal and plastic aggregates and type of substitution are provided. 

The comparisons in Figure 12 highlight a high scatter in the results. In general, it can be observed that introducing fine plastic aggregates into concrete mixtures reduces the strength, as noted by Almeshal et al. [20], who introduced crushed PET bottles with a particle size of 4–0.075 mm into a concrete mix at various percentages. When plastic aggregates with larger sizes (8–20 mm) were inserted into the mix, a significant decrease in strength occurred, as discussed by Belmokaddem et al. [23]. Moreover, when the percentage of plastic aggregates is equal to or less than 10%, the decrease in strength is relatively minor, typically less than 20%. However, when the percentage of plastic aggregates reaches or exceeds 30%, a more significant loss in strength is observed, exceeding 50%. Another important observation is that the nature of the plastic can significantly contribute to the variation in strength. As observed in [23], the same percentage of plastic aggregates added to the same control mix can lead to different results in strength. This is likely related to the mass density of the plastic material. In [23], the different effects of plastics with different true mass densities were observed, similar to those observed in this study. However, in the present case, we examined what influenced the overall mass density of the mix. Obviously, introducing plastics with a higher mass density causes a lower reduction in the mass density of the mix. This is probably the parameter that influences the variation in strength. In the present study, C1P2 mix had the lowest mass density (Figure 13) and thus experienced the highest variation in strength. In ref. [20], the dimensions of the plastic particles in mix C1P2 were in the range of 5–20 mm, and, consistent with what was discussed in [20], where plastic particles with a size lower than 4 mm were used, the high variation in strength may have been due to the size of the plastic particles. The locution “may be” is essentially because, in the case of mix C2P3, where plastic granules with a nominal dimension of 10 mm were used, the effect of size on the variation in strength was far from evident. What is common to the experimental campaigns where different mixes characterized by different plastic aggregates were analyzed (present study, [23]) is the dependence of the variation in strength on the mass density of the mix. Unfortunately, this is not consistent when experimental campaigns by different authors are compared: the reference mix in ref. [23] had a mass density of 2290 kg/m^3^, and when the strength was reduced to 40%, the mass density was 1722 kg/m^3^ (obtained with the introduction of 40% plastics by true volume. In ref. [20], where the reference mix had a mass density of 2420 kg/m^3^, the reduction in strength by 40% was obtained with a plastic percentage lower than 10% by true volume and a mass density higher than 2200 kg/m^3^, a value that is very close to the mass density of the reference mix in [23]. 

These results, once again, suggest the need to support experimental campaigns devoted to deeply understanding the mechanisms of the interaction of plastics with the classical components of concrete.

The comparisons in Figure 14 pertain to the introduction of polypropylene fibers to concrete mixtures. In this case, the reduction in strength is less evident, likely due to the introduction of a small percentage of plastic in the mix (less than 0.65% by weight). In some cases, the introduction of polypropylene fibers led to an increase in strength of up to 20%, as observed by Siddique et al. [15]. Moreover, it is interesting to note that for the same concrete mix and percentage of plastic fibers, a potential discriminating factor is the length of the fiber. In general, the results in the literature show that increasing the compressive strength using plastic fibers in the mix is possible but, once again, the results are controversial, revealing that the parameters involved are more than the percentage, the type, and the length of fibers. 

## 5. Conclusions

In recent decades, the emergence of plastic pollution has created numerous challenges, prompting the need for innovative strategies for plastic waste disposal. Against this background, the construction sector has emerged as a promising avenue, with researchers exploring the possibility of directly recycling plastic waste by incorporating it into concrete as aggregates. Consequently, significant efforts have been directed toward this strategy.

Generally, the introduction of plastic waste into concrete leads to reductions in mechanical performance and workability. Conversely, it imparts valuable properties such as light weight as well as thermal and acoustic insulation. This makes concrete containing plastics a material that is well suited for nonstructural and lightweight elements within construction projects.

The present study, investigating the potential of incorporating plastics into concrete mixes, discussed the controversial data in the literature regarding the effect of plastics’ incorporation on the mechanical characteristics of concrete and the need to obtain new data from experimental investigations. 

Two ordinary concrete mixes and three types of normal concrete were considered, with different types, sizes, and percentages of plastic added to the mixture. Also, the introduction of polypropylene fibers in pervious concrete was investigated to improve the data in the literature for this type of concrete. In the latter case, the effect of fibers was studied in mixes with and without pozzolanic admixtures such as fly ash and silica fume.

The experimental campaign revealed the following:

(1) There is a dependence of the concrete on the mass density of the reduction in strength with increasing percentage of plastic aggregates;
(2) Reference mixes characterized by a lower mass density featured a larger variation in compressive strength;
(3) There was no clear dependence of the variation in concrete strength on the size of the plastic aggregates but there was a dependence on the true mass density of the plastic aggregate for the resultant mass density of concrete;
(4) Low mass density concrete (lower than 2100 kg/m^3^) cannot receive more than 20% of true volume of plastic aggregates if one wants to guarantee 40% of the strength of the reference mix.
(5) High-mass-density concrete (higher than 2400 kg/m^3^) can contain up to 30% of the true volume of plastic aggregates to guarantee 40% of the strength of the reference mix;
(6) The results in bullets 4 and 5 do not depend on the strength of the reference concrete in the range of 20–40 MPa.

The comparisons with some experimental campaigns in the literature revealed the following:

(1) The amount of the variation in the mass density associated with a variation in the normalized compressive strength in different experimental campaigns is inconsistent.
(2) Mixes with higher mass densities may have a much higher variation in the strength with the same percentage of added plastic aggregates.
(3) Increasing the dimension of the plastic aggregates is not consistently associated with a larger variation in the concrete strength.
(4) These observations show that further experimental campaigns need to be conducted to determine rules for practical applications.

Furthermore, the inclusion of plastic fibers in pervious concretes results in less pronounced variations in compressive strength even if, in some cases, mixes containing plastic fibers exhibit higher compressive strength than the control mixes. These findings are attributed to the relatively low percentages of plastic fibers added to the mixes, as well as the contribution provided by the fibers too reducing the Poisson effect and microcrack propagation. However, introducing high percentages of plastic fibers can lead to segregation problems during mixing, which requires further study to solve. Furthermore, in this case as well, more experimental data are needed to definitively determine the mechanisms involved, considering the high number of variables involved.

In conclusion, the careful consideration of the type, size, and proportion of plastics is essential to leverage the benefits of integrating waste plastic into concrete effectively. Further studies are needed to determine the optimal additive percentages and sustainable methods for assessing the influence of the size of waste plastics, thereby improving the feasibility of using recycled plastic in construction applications.

## Figures and Tables

**Table 1:** Concrete mixes.

Concrete Type	Plastic Aggregate		Sample Group ID
	Type	Bulk Volume Percentage	
C1—concrete with medium– low strength (20 MPa)	P1—light blue plastic flakes	-	Ref. C1_P1_0%
		10%	C1_P1_10%
		20%	C1_P1_20%
		30%	C1_P1_30%
C2—concrete with medium– high strength (40 MPa)	P1—light blue plastic flakes	-	Ref. C2_P1_0%
		2%	C2_P1_2%
		4%	C2_P1_4%
		6%	C2_P1_6%
		30%	C2_P1_30%
C1—concrete with medium– low strength (20 MPa)	P2—plastic flakes	-	Ref. C1_P2_0%
		10%	C1_P2_10%
		20%	C1_P2_20%
C2—concrete with medium– high strength (40 MPa)	P3—plastic granules	-	Ref. C2_P3_0%
		10%	C2_P3_10%
		20%	C2_P3_20%
		30%	C2_P3_30%
PC—pervious concrete	FPP—plastic fibers	-	Ref. PC_FPP_0%
		0.2%	PC_FPP_0.2%
PC-SF—pervious concrete with silica fume	FPP—plastic fibers	-	Ref. PC-SF_FPP_0%
		0.2%	PC-SF_FPP_0.2%
PC-FA—pervious concrete with fly ash	FPP—plastic fibers	-	Ref. PC-FA_FPP_0%
		0.2%	PC-FA_FPP_0.2%

**Table 2:** Average experimental results for concrete mixes C1 and C2.

Sample Group ID	Compressive Strength [MPa]			Normalized Compressive Strength [-]		
	7 Days	14 Days	28 Days	7 Days	14 Days	28 Days
Ref. C1_P1_0%	17.0	20.1	22.6			
C1_P1_10%	14.2	16.7	18.6	0.83	0.83	0.82
C1_P1_20%	11.2	13.3	14.8	0.66	0.66	0.65
C1_P1_30%	9.0	10.8	11.8	0.53	0.54	0.52
Ref. C2_P1_0%	33.3	36.1	39.6			
C2_P1_2%	29.5	33.1	36.3	0.89	0.92	0.92
C2_P1_4%	28.9	30.5	33.4	0.87	0.85	0.84
C2_P1_6%	26.5	28.9	31.5	0.79	0.80	0.80
C2_P1_30%	15.1	17.9	19.6	0.45	0.50	0.50
Ref. C1_P2_0%	13.0	16.0	18.5			
C1_P2_10%	10.3	11.7	13.0	0.79	0.73	0.70
C1_P2_20%	6.5	8.0	8.6	0.50	0.50	0.46
Ref. C2_P3_0%	34.0	36.0	39.4			
C2_P3_10%	24.5	28.2	31.7	0.72	0.78	0.81
C2_P3_20%	20.9	23.6	26.3	0.61	0.66	0.67
C2_P3_30%	12.5	13.9	15.3	0.37	0.39	0.39

**Table 3:** Average experimental results for pervious concrete mixes PC, PC-SF, and PC-FS.

Sample Group ID	Compressive Strength [MPa]			Normalized Compressive Strength [-]		
	7 Days	28 Days	90 Days	7 Days	28 Days	90 Days
Ref. PC_FPP_0%	21.0	28.8	n.a.			
PC_FPP_0.2%	27.1	30.0	31.9	1.29	1.04	n.a.
Ref. PC-SF_FPP_0%	28.2	34.0	36.6			
PC-SF_FPP_0.2%	28.3	32.7	34.3	1.00	0.96	0.94
Ref. PC-FA_FPP_0%	33.6	38.4	39.5			
PC-FA_FPP_0.2%	30.3	34.6	35.0	0.90	0.90	0.89

n.a. = not available.

## Data Availability

The original contributions presented in the study are included in the article, further inquiries can be directed to the corresponding author.
